# New York-American Veterinary College

**Published:** 1901-07

**Authors:** 


### COMMENCEMENT EXERCISES.

### NEW YORK-AMERICAN VETERINARY COLLEGE.

The New York University held its sixty-ninth annual commencement exercises at the Opera House on June 6, 1901.

The veterinary candidates for diplomas were presented by William T. Coates, M.D., D.V.S., Secretary, and the degree of Doctor of Veterinary Surgery was conferred upon the following gentlemen ; Robert Girard Bose, Troy, N. Y. ; David Samuel Johnson, New York, N. Y. ; Charles Joseph Jones, A.B., New York, N. Y.; Rudolph Frederick Meiners, Rutherford, N. J. ; Franklin Stern Morris, M.E., Bucksville, Pa. ; Isaac Wertheimer, New York, N. Y.

The following passed the examinations, but will not receive the degree until they have complied with the Regents' requirements : William C. Miller, New York, N. Y. ; J. S. Serling, New York, N. Y. ; F. H. Werner, New York, N. Y.

The following prizes were awarded : Faculty prize, Franklin Stern Morris, M.E., for the best general examination, a gold medal. Alumni prize : William C. Miller, for the second best general examination, a set of text-books.

